# A Closed-Loop Clinical Audit on Compliance With National Institute for Health and Care Excellence (NICE) Guidelines (NG232) for CT Cervical Spine Imaging in Adult Head Injury Patients

**DOI:** 10.7759/cureus.104620

**Published:** 2026-03-03

**Authors:** Ismail Aslam, Tasleem Abbas, Junaid Mubashir

**Affiliations:** 1 Neurological Surgery, Imran Idrees Teaching Hospital, Sialkot, PAK

**Keywords:** audit and re-audit cycle, audit in tertiary care hospital, cervical spine imaging, cervical spine injury, ct scan, head injury, nice guidelines

## Abstract

Objective: CT cervical spine imaging is essential for early detection of cervical spine injuries in adult head trauma patients. Standard clinical guidelines provide specific criteria for when CT cervical spine imaging should be performed in such patients. This audit aimed to assess and improve compliance with these guidelines in a tertiary care neurosurgical unit in Pakistan. Methods: A closed-loop clinical audit was conducted at Imran Idrees Teaching Hospital, Sialkot. The initial audit retrospectively assessed adult head injury patients against the recommended guidelines criteria. A targeted intervention was implemented, including staff education, dissemination of guideline posters, and reinforcement of documentation practices. A prospective re-audit was then performed, and compliance before and after the intervention was compared. Results: In the initial audit cycle, 34 out of 37 patients met the guideline criteria, of whom 10 (29.4%) underwent CT cervical spine imaging. Following the intervention, 30 out of 33 patients met the criteria in the re-audit, and 25 (83.3%) of them received appropriate imaging. The intervention effectively improved guideline adherence and closed the audit loop.

Conclusion: This closed-loop audit demonstrated that targeted interventions can significantly improve compliance with standard clinical guidelines for cervical spine imaging in head injury patients. Regular audit cycles, staff education, and guideline reinforcement play a key role in enhancing imaging practices and patient safety, especially in low-resource healthcare settings.

## Introduction

Head trauma is a common presentation in emergency departments and is often associated with cervical spine injury, particularly in patients with reduced consciousness, neurological deficits, or high-risk mechanisms of injury [1]. Patients with traumatic brain injury are at increased risk of cervical spine injury compared to those with non-head-related blunt trauma [2]. Failure to recognize unstable cervical injuries during the initial evaluation may result in serious neurological deterioration, highlighting the importance of timely and appropriate imaging [3]. Early and accurate detection of cervical spine injuries is crucial for preventing secondary spinal cord damage and guiding appropriate clinical management [4].

CT is significantly more sensitive than plain radiography in detecting cervical spine injuries in trauma patients and is particularly effective in obtunded or intubated individuals, where its superior diagnostic accuracy makes it the preferred first-line imaging modality [1,5].

To address this, the National Institute for Health and Care Excellence (NICE) published guideline NG232 [6], which outlines clear indications for performing CT cervical spine imaging in adult patients with head injury. These criteria aim to ensure imaging is used appropriately and consistently in trauma care pathways. Despite the availability of such evidence-based guidelines, compliance in clinical practice can vary, particularly in low-resource settings. Factors such as limited awareness of guidelines, high workload in emergency departments, and inconsistent documentation can contribute to suboptimal adherence [7,8].

This audit was conducted to evaluate compliance with NICE NG232 guidelines [6] for CT cervical spine imaging in adult head injury patients at a tertiary care neurosurgical unit in Pakistan. Following the initial audit, targeted interventions were implemented, including staff education sessions, visual displays of guideline summaries in clinical areas, and reinforcement of documentation practices to improve awareness and adherence to standard guidelines. A re-audit was subsequently performed to assess the effectiveness of these measures and to close the audit loop.

## Materials and methods

Setting and study design

This closed-loop clinical audit was conducted in the neurosurgery department and emergency department of Imran Idrees Teaching Hospital, a tertiary care center located in Sialkot, Pakistan. The audit evaluated adherence to the National Institute for Health and Care Excellence (NICE) guideline NG232 [6], which outlines indications for CT cervical spine imaging in adult patients with head injury. The audit was conducted in two cycles. The initial audit was retrospective, covering the period from October 1 to November 30, 2024. This was followed by an intervention period in December 2024. The re-audit was prospective and took place from January 1 to February 28, 2025.

Inclusion and exclusion criteria

This study included patients aged 16 years and older who presented with a head injury and were admitted either through the emergency department or the neurosurgery department. Patients were eligible for inclusion if they met the indications for cervical spine imaging as per the NG232 criteria [6]. Exclusion criteria comprised patients under the age of 16, those with incomplete documentation that prevented application of the NG232 criteria [6], and patients referred from other centers who had already undergone cervical spine imaging.

Audit standards

The audit was based on the NICE NG232 guideline, specifically recommendations 1.6.2 and 1.6.3, which outline criteria for performing CT cervical spine imaging in adult patients with head injury [6].

According to recommendation 1.6.2, a CT cervical spine scan should be performed within one hour in patients aged 16 and over who have sustained a head injury, if any high-risk criteria are present. These include a Glasgow Coma Scale (GCS) [9] score of 12 or less on initial assessment, intubation, urgent need for a definitive cervical spine diagnosis (e.g., before surgery), or blunt polytrauma involving the head and other major body regions. Additionally, CT is indicated when there is clinical suspicion of cervical spine injury along with one or more of the following: age 65 years or older, high-risk mechanism of injury (such as a fall from a height >1 meter or 5 stairs, axial load to the head, high-speed or rollover motor vehicle collision, ejection from a vehicle, or involvement of motorized recreational vehicles or bicycle collision), focal neurological deficit, or paraesthesia in the upper or lower limbs.

Recommendation 1.6.3 advises CT cervical spine imaging for patients with head injury and neck pain or tenderness who do not meet the high-risk criteria above, but exhibit one or more of the following: inability to safely assess the range of neck movement, inability to actively rotate the neck 45 degrees to the left and right, or presence of conditions predisposing to higher risk of cervical spine injury (such as axial spondyloarthritis).

Data collection

For the initial audit cycle, data were collected retrospectively by reviewing emergency admission notes and radiology logs. In contrast, the re-audit cycle involved prospective data collection using a structured proforma (see the Appendices) specifically designed to capture key clinical and imaging parameters. For each patient, the following information was documented: age, gender, GCS [9] score on presentation, mechanism of injury, presence or absence of neck pain, and whether the patient met any NG232 guideline criteria [6] for CT cervical spine imaging. Additionally, the performance of CT cervical spine imaging was recorded, along with the imaging result (e.g., normal findings, cervical spine fracture, or other abnormalities) and the final clinical outcome, including whether the patient was admitted, underwent surgery, or was discharged.

Intervention

In December 2024, a targeted intervention was implemented to improve adherence to the NICE NG232 guidelines [6]. This included structured educational sessions conducted for emergency medicine and neurosurgery doctors to enhance awareness and understanding of the CT cervical spine imaging criteria. In addition, visual aids such as posters summarizing the NG232 recommendations [6] were displayed prominently in high-traffic clinical areas, including the emergency department and neurosurgery ward. Furthermore, clinicians were encouraged to explicitly document the rationale for imaging decisions in patient notes, reinforcing guideline-based clinical reasoning and accountability in daily practice.

Outcome measure

The primary outcome was the compliance rate with NICE NG232 guidelines [6], defined as the proportion of patients who met the criteria and appropriately received CT cervical spine imaging.

Ethical approval

This audit and the subsequent re-audit were approved by the Institutional Review Board (IRB) of Sialkot Medical College, with which Imran Idrees Teaching Hospital is affiliated.

## Results

Initial audit cycle

**Figure 1:**
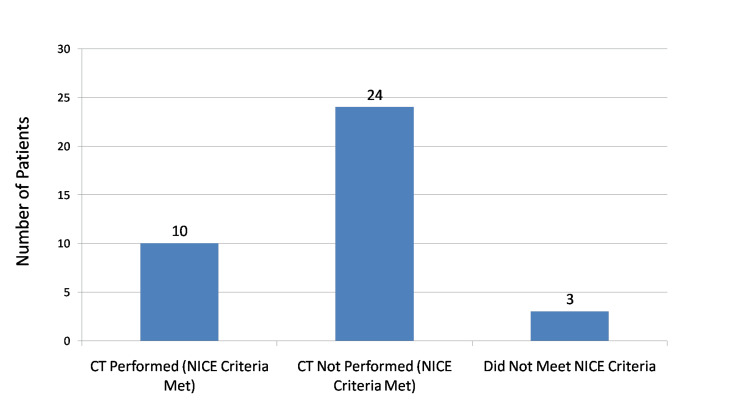
Initial audit results: CT cervical spine imaging compliance in adult head injury patients (October–November 2024) NICE: National Institute for Health and Care Excellence

During the initial audit cycle (October 1 to November 30, 2024), a total of 37 adult head injury patients were included. Of these, 34 patients met the NICE NG232 criteria [6] for CT cervical spine imaging. However, only 10 (29.4%) patients who met the criteria underwent CT cervical spine imaging, while 24 (70.6%) eligible patients did not receive appropriate imaging. The remaining three patients did not meet the NICE criteria [6] and were appropriately not scanned. The findings from the initial audit cycle are shown in Figure 1.

Re-audit cycle

**Figure 2:**
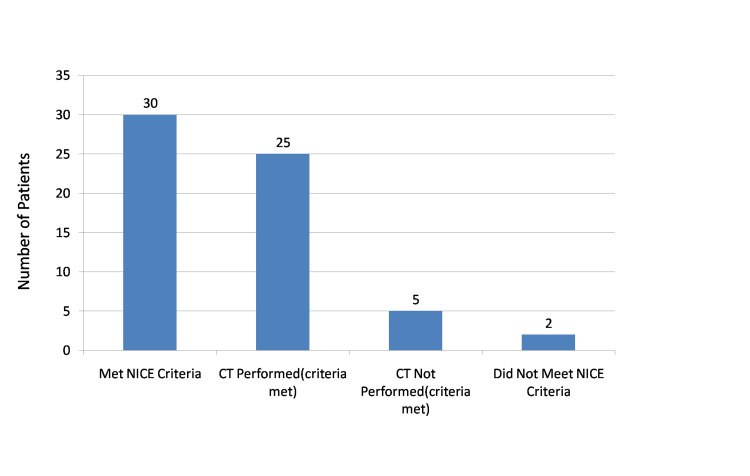
Re-audit results: CT cervical spine compliance in adult head injury patients (January 1 to February 28, 2025)

In the re-audit cycle (January 1 to February 28, 2025), 32 patients were included. Among these, 30 met the NG232 criteria [6], and 25 (83.3%) of them underwent CT cervical spine imaging, while five (16.7%) eligible patients did not. The remaining two patients did not meet NG232 criteria [6]. This demonstrated a marked improvement in compliance following the intervention phase. The re-audit cycle results are presented in Figure 2.

The improvement from 29.4% (10/34) in the initial audit to 83.3% (25/30) in the re-audit was statistically significant (p < 0.0001). The absolute increase in compliance was 53.9%, with a 95% confidence interval for the absolute difference of 31.0% to 76.8 (calculated using the Newcombe-Wilson method). 

**Figure 3:**
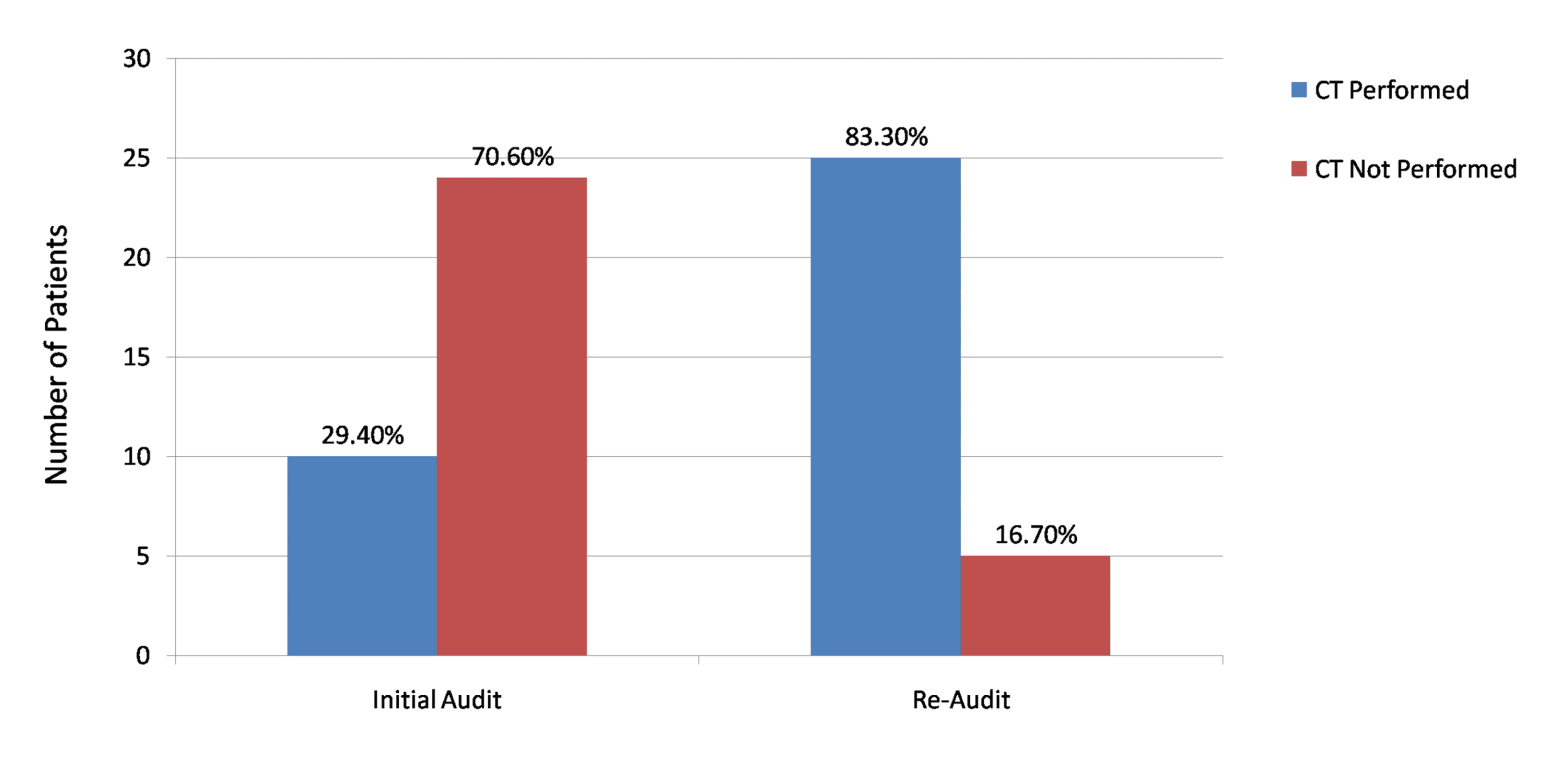
Comparison of CT cervical spine imaging compliance in adult head injury patients: initial audit vs re-audit

The increase in compliance was attributed to the targeted interventions carried out in December 2024, including staff education, visual dissemination of guideline criteria, and reinforcement of proper documentation practices. Additionally, improvements were observed in clinical documentation of GCS [9] score, mechanism of injury, symptoms suggestive of cervical spine injury, and criteria-based decision-making during the re-audit period. A comparative analysis of initial audit and re-audit findings is illustrated in Figure 3, highlighting the improvement in guideline compliance following intervention.

## Discussion

This closed-loop clinical audit demonstrated a significant improvement in compliance with NICE guideline NG232 [6] for CT cervical spine imaging in adult head injury patients. The baseline audit revealed a low compliance rate of 10/34 (29.4%), highlighting a substantial gap between recommended practice and clinical implementation. After targeted interventions, including staff education and visual reinforcement of the guidelines, compliance improved markedly to 25/30 (83.3%) in the re-audit cycle. The improvement in adherence reflects the effectiveness of simple, low-cost quality improvement strategies, such as guideline dissemination, staff engagement, and documentation reinforcement [7]. These interventions are particularly valuable in low-resource settings, where systemic challenges such as high patient volume, limited radiology access, and inconsistent documentation can hinder the implementation of evidence-based protocols [8].

The importance of adhering to established trauma protocols, such as BOAST [10], ATLS [11], and NICE [6], is well documented. These protocols ensure early identification and management of cervical spine injuries, reduce the risk of secondary neurological damage, and improve patient outcomes in trauma settings [12]. Studies have reported that approximately one-third of patients with cervical spine and/or spinal cord injuries also sustain moderate to severe head injuries. Notably, upper cervical spine injuries are more frequently associated with severe intracranial pathology, including skull base fractures and hematomas, highlighting the critical need for early cervical spine assessment in head-injured patients [13].

The likelihood of cervical spine injury increases with the severity of head trauma, underscoring the need for systematic evaluation, safe transport, and careful management of trauma patients to prevent devastating consequences if such injuries are missed or mismanaged [14]. Prior research has highlighted that patients with clinically significant head injuries are at a substantially higher risk of cervical spine injuries compared to those without head trauma [2]. Additionally, evidence from large cohort studies has demonstrated that the incidence of cervical spine injury is significantly elevated in patients presenting with a GCS [9] score of 8 or lower, emphasizing the critical role of early imaging in this vulnerable group [15]. Practice management guidelines from the Eastern Association for the Surgery of Trauma further reinforce the importance of timely identification of cervical spine injuries in trauma settings, supporting the use of standardized imaging protocols [16].

NICE NG232 [6] provides clear and objective criteria for when CT cervical spine imaging should be performed, aiming to optimize patient safety while avoiding unnecessary radiation exposure and resource use [5]. CT has been shown to be significantly more sensitive than plain radiography in detecting cervical spine injuries in trauma patients, supporting its use as the first-line modality [1]. In our setting, lack of awareness and inconsistent application of these criteria were likely contributors to the low baseline compliance rate. By addressing these factors through targeted education and system-level adjustments, our audit successfully closed the loop and improved imaging practices.

This audit also demonstrated improvement in documentation of key clinical indicators, including GCS [9] score, mechanism of injury, and symptoms suggestive of cervical spine injury. Better documentation supports clinical decision-making, facilitates audit processes, and enhances medicolegal safety [7].

Limitations

While this audit was conducted at a single tertiary care center with a modest sample size, it demonstrated a clear and clinically meaningful improvement in compliance with established cervical spine imaging protocols. The findings may not be generalizable to other institutions with different patient demographics, resources, or clinical workflows. Additionally, the potential influence of the Hawthorne effect cannot be entirely excluded.

## Conclusions

This closed-loop clinical audit demonstrated that adherence to NICE NG232 guidelines for CT cervical spine imaging in adult head injury patients can be significantly improved through targeted interventions. These included structured teaching sessions for emergency and neurosurgery staff, visual displays of the NG232 criteria in key clinical areas such as the ED (including the trauma bay) and neurosurgical ward, and verbal reinforcement of the importance of proper documentation during clinical rounds. The increase in compliance from 10/34 (29.4%) to 25/30 (83.3%) highlights the impact of simple quality improvement measures in enhancing patient care and imaging safety. Regular audit cycles and continuous staff engagement are essential for maintaining high standards of guideline-based clinical practice, particularly in resource-limited healthcare settings.

## Appendices

Proforma

Patient Information - Audit ID Number: ___________ - Date of Admission: ___________ - Age: _____ - Gender: ☐Male  ☐ Female Injury Details - Date and Time of Injury: _________________ - Mechanism of Injury: ___________________ - Glasgow Coma Scale (GCS) at Presentation: E___ V___ M___ =____ /15 Clinical Findings - Neck Pain or Tenderness  ☐ Yes ☐ No - Neurological Deficit  ☐ Yes ☐ No - Post-Traumatic Amnesia ☐ Yes ☐ No - Focal Neurological Signs ☐ Yes ☐ No - Intoxication/Suspected Intoxication ☐ Yes ☐ No - Age ≥ 65 years ☐ Yes ☐ No - GCS < 12 on initial assessment ☐ Yes ☐ No - Other Relevant Clinical Notes: _____________________________ Imaging Details - CT Cervical Spine Performed ☐Yes   ☐ No - Date of CT: ___________ - CT Findings: o ☐ Normal o ☐ Cervical Spine Fracture o ☐ Other (please specify): __________________________

Outcome - Disposition: ☐ Taken to Surgery  ☐ Admitted  ☐ Discharged

- Final Diagnosis: ________________________________________

Auditor’s Initials: __________ Date of Data Entry: __________

## References

[1] Holmes JF, Akkinepalli R (2005). Computed tomography versus plain radiography to screen for cervical spine injury: a meta-analysis. J Trauma.

[2] Hills MM, Deanne SA (1993). Head injury and facial injury: is there an increased risk of cervical spine injury?. J Trauma.

[3] Holly LT, Kelly DF, Counelis GJ, Blinman T, McArthur DL, Cryer HG (2002). Cervical spine trauma associated with moderate and severe head injury: incidence, risk factors, and injury characteristics. J Neurosurg.

[4] Stiell IG, Wells GA, Vandemheen KL (2001). The Canadian C-spine rule for radiography in alert and stable trauma patients. JAMA.

[5] Panczykowski DM, Tomycz ND, Okonkwo DO (2011). Comparative effectiveness of using computed tomography alone to exclude cervical spine injuries in obtunded or intubated patients: meta-analysis of 14,327 patients with blunt trauma. J Neurosurg.

[6] (2026). Head injury: assessment and early management. March 22.

[7] Scally G, Donaldson LJ (1998). The NHS's 50 anniversary. Clinical governance and the drive for quality improvement in the new NHS in England. BMJ.

[8] Jacobson MA, Ryan D (2014). Audit into the appropriateness of CT cervical spine scan requesting in the emergency department. Res Medica.

[9] Teasdale G, Jennett B (1974). Assessment of coma and impaired consciousness. A practical scale. Lancet.

[10] (2026). BOASt - cervical spine clearance in the trauma patient. May.

[11] American College of Surgeons Committee on Trauma (2026). Advanced Trauma Life Support (ATLS®). ACS ATLS overview page.

[12] Ahmed MM, Kiani S, Shah MT, El-Zanaty A, Ali MS (2025). Early management of suspected cervical spine injury: a real-world insight from a London major trauma centre. Cureus.

[13] Iida H, Tachibana S, Kitahara T, Horiike S, Ohwada T, Fujii K (1999). Association of head trauma with cervical spine injury, spinal cord injury, or both. J Trauma.

[14] Nazir M, Khan SA, Raja RA, Bhatti SN, Ahmed E Cervical spinal injuries in moderate to severe head injuries. J Ayub Med Coll Abbottabad.

[15] Paiva WS, Oliveira AM, Andrade AF, Amorim RL, Lourenço LJ, Teixeira MJ (2011). Spinal cord injury and its association with blunt head trauma. Int J Gen Med.

[16] Como JJ, Diaz JJ, Dunham CM (2009). Practice management guidelines for identification of cervical spine injuries following trauma: update from the eastern association for the surgery of trauma practice management guidelines committee. J Trauma.

